# Crystal structure of (C_9_H_17_N_2_)_3_[Bi_2_I_9_]

**DOI:** 10.1107/S2056989021007799

**Published:** 2021-08-06

**Authors:** Zoe James, Yunhe Cai, Paz Vaqueiro

**Affiliations:** a Department of Chemistry, University of Reading, Whiteknights, Reading, RG6 6DX, UK

**Keywords:** crystal structure, iodo­bis­muthate, photovoltaic materials

## Abstract

Single crystals of tris­(2,3,4,6,7,8,9,10-octa­hydro­pyrimido[1,2-*a*]azepin-1-ium) tri-μ_2_-iodido-bis­[tri­iodido­bis­muth(III)], (C_9_H_17_N_2_)_3_[Bi_2_I_9_], were prepared by a solvothermal method, heating a mixture of BiI_3_, KI, 1,8-di­aza­bicyclo­[5.4.0]undec-7-ene (DBU) and ethanol at 443 K for six days. The asymmetric unit of the title compound, which crystallizes in the monoclinic space group *P*2_1_/*c*, contains one [Bi_2_I_9_]^3−^ anion and three protonated DBUH^+^ moieties. The dinuclear [Bi_2_I_9_]^3−^ anions, which are composed of face-sharing BiI_6_^3−^ octa­hedra, are packed in columns parallel to the [010] direction, and separated by protonated DBUH^+^ moieties. The optical band gap of (C_9_H_7_N_2_)_3_Bi_2_I_9_ is 2.1 eV.

## Abstract 2

The crystal structure of (C_9_H_7_N_2_)_3_Bi_2_I_9_ contains dinuclear [Bi_2_I_9_]^3−^ anions, formed by two face-sharing octa­hedra, and protonated 1,8-di­aza­bicyclo­[5.4.0]undec-7-ene (DBU) cations.

## Chemical context   

In recent years, hybrid bis­muth halides have attracted considerable attention owing to their inter­esting physical properties (Adonin *et al.*, 2016 ▸), including luminescence (Adonin *et al.*, 2015 ▸), non-linear optical effects (Bi *et al.*, 2008 ▸) and thermochromism (García-Fernández *et al.*, 2018 ▸). Moreover, the lack of stability and the toxicity of lead halide perovskites has stimulated research efforts into bis­muth-containing halides as stable and environmentally friendly alternatives for photovoltaic applications (Wu *et al.*, 2020 ▸). However, bis­muth-containing hybrid halides, such as (CH_3_NH_3_)_3_Bi_2_I_9_ (Eckhardt *et al.*, 2016 ▸), often adopt low-dimensional structures, which in most cases result in larger band gaps than those of lead halide perovskites (Wang *et al.*, 2020 ▸). Examples of two- or three-dimensional structures are very rare, and include a two-dimensional metal-deficient perovskite, (H_2_AEQT)Bi_2/3_I_4_ (AEQT = 5,5′′′’-bis­(amino­eth­yl)-2,2′:5′,2′′:5′′,2′′′-quaterthio­phene) (Mitzi, 2000 ▸) and the two-dimensional mixed halide (TMP)_1.5_[Bi_2_I_7_Cl_2_]_4_ (TMP = *N*,*N*,*N*′,*N′*-tetra­methyl­piperazine) (Li *et al.*, 2017 ▸). The vast majority of hybrid bis­muth halides contain zero-dimensional units, which are either discrete polynuclear or mononuclear anionic units, depending on the synthetic conditions and the nature of the organic counter-cations (Wu *et al.*, 2009 ▸). In these materials, the Bi^3+^ cation typically adopts a distorted octa­hedral coordination, either forming mononuclear anions or polynuclear anions in which octa­hedra share edges or faces. Dinuclear species, such as [Bi_2_I_8_]^2−^, [Bi_2_I_9_]^3−^ and [Bi_2_I_10_]^4−^, are one of the most widespread types of bis­muth-halide units (Adonin *et al.*, 2016 ▸).

## Structural commentary   

**Figure 1:**
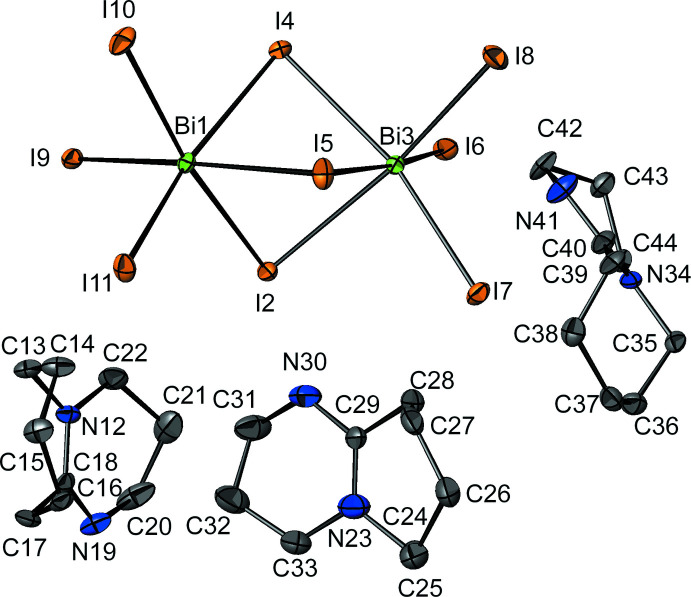
The asymmetric unit of (C_9_H_7_N_2_)_3_Bi_2_I_9_ with displacement ellipsoids drawn at the 50% probability level. H atoms have been omitted for clarity. Only one orientation of the disordered DBUH^+^ ring is shown.

The asymmetric unit of (C_9_H_7_N_2_)_3_Bi_2_I_9_ comprises three protonated DBUH^+^ cations, one of which is disordered (see *Refinement*), and one [Bi_2_I_9_]^3−^ anion (Fig. 1 ▸). The two Bi^3+^ cations found in the [Bi_2_I_9_]^3−^ unit are octa­hedrally coord­inated by six iodides, with Bi—I distances ranging between 2.9532 (4) and 3.2788 (4) Å. Each BiI_6_^3−^ octa­hedron shares one face with a second octa­hedron, forming a dinuclear unit, [Bi_2_I_9_]^3−^. The Bi—I distances for the bridging μ_2_-I^−^ anions, which range between 3.1405 (5) and 3.2788 (4) Å, are significantly longer than those for the terminal iodides [2.9532 (4) to 2.9908 (5) Å]. The angles for Bi^3+^—μ_2_-I^−^—Bi^3+^ range from 78.144 (9) to 80.095 (10)°, while those for terminal I^−^—Bi ^3+^—μ_2_-I^−^ take values between 85.283 (13) and 97.725 (12)°. The face-sharing arrangement of BiI_6_^3−^ octa­hedra and the distances and angles are similar to those found in other compounds containing [Bi_2_I_9_]^3−^ anions, such as (CH_3_NH_3_)_3_Bi_2_I_9_ (Eckhardt *et al.*, 2016 ▸) or (C_3_H_5_N_2_)_3_Bi_2_I_9_ (Węcławik *et al.*, 2016 ▸).
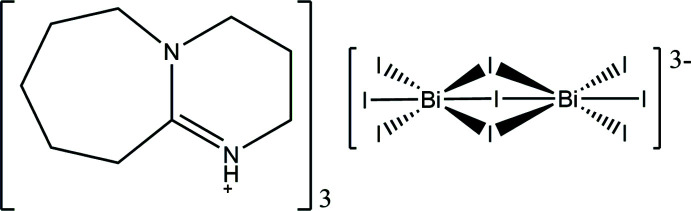


## Supra­molecular features   

**Figure 2:**
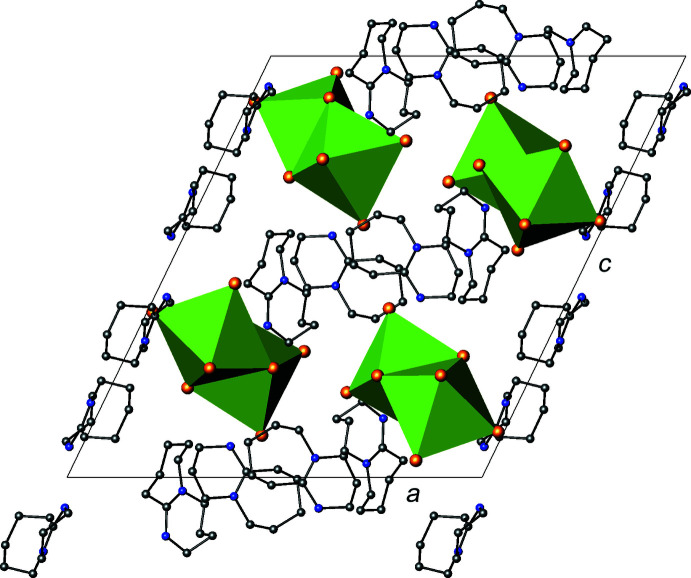
View of the packing of the title compound along [010]. Key: bis­muth, green polyhedra; iodine, large orange spheres; carbon, small grey spheres; nitro­gen, small blue spheres. H atoms have been omitted for clarity.

The [Bi_2_I_9_]^3−^ dinuclear units are packed in columns parallel to the [010] direction (Fig. 2 ▸), separated by the protonated DBUH^+^ cations. There are no short I⋯I distances between the [Bi_2_I_9_]^3−^ anions, implying that there are limited inter­actions that could lead to extended electronic delocalization.

**Figure 3:**
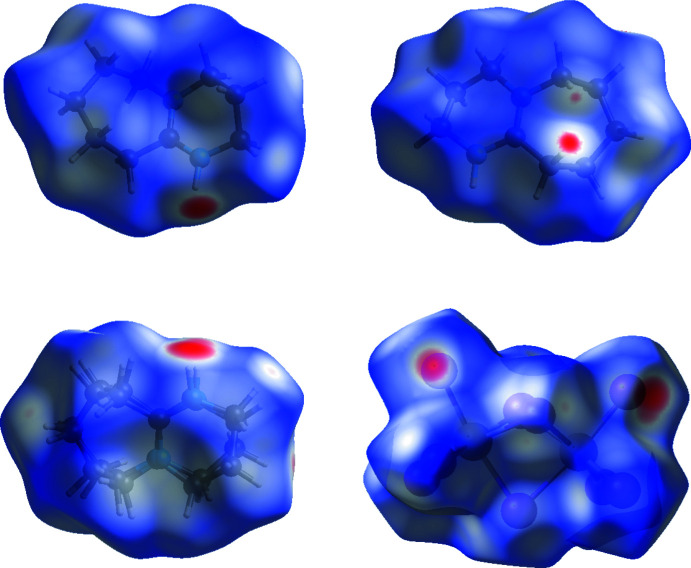
Hirshfeld surfaces, mapped with *d*_norm_, of the three crystallographically independent DBUH^+^ cations and the [Bi_2_I_9_]^3−^ anion. The red areas correspond to regions where contacts are shorter than the sum of the van der Waals radii.

As shown in Table 1 ▸, there is hydrogen bonding between the amine functional groups in the DBU moieties and the [Bi_2_I_9_]^3−^ dinuclear units. It should be noted that H30 does not form a hydrogen bond. This may be related to the fact that N30 is almost equidistant to I2, I5, I7 and I11. In addition, there are also short contacts of the type C—H⋯I. Hirshfeld surface analysis was performed using *Crystal Explorer 17* (Turner *et al.*, 2017 ▸), with standard resolution of the *d*_norm_ surfaces. A number of short H⋯I contacts are highlighted in red in the Hirshfeld surfaces for the DBU cations and the [Bi_2_I_9_]^3−^ anion (Fig. 3 ▸). Examination of the fingerprint plots for the DBUH^+^ cations (see supporting information), resolved into H⋯H and H⋯I contacts, reveals that approximately 30% of the surface area corresponds to H⋯I contacts, with the remaining area corresponding to H⋯H inter­actions.

## Database survey   

A search in the Cambridge Structural Database (CSD Version 2020.3, December 2020; Groom *et al.*, 2016 ▸) reveals that there are numerous compounds containing the dinuclear [Bi_2_I_9_]^3−^ anion, also found in the compound reported here. This includes examples in which the counter-cation is an organic moiety, such as (CH_3_NH_3_)_3_Bi_2_I_9_ (Eckhardt *et al.*, 2016 ▸) or (C_3_H_5_N_2_)_3_Bi_2_I_9_ (Węcławik *et al.*, 2016 ▸), but also compounds in which the counter-cation is a transition-metal or a rare-earth complex, such as [Co(C_2_H_8_N_2_)_3_][Bi_2_I_9_] (Goforth *et al.*, 2005 ▸) or [*Ln*(DMF)_8_][Bi_2_I_9_] (*Ln* = Y, Tb) (Mishra *et al.*, 2012 ▸). The ubiquitous dinuclear [Bi_2_I_9_]^3−^ anion has also been found in compounds containing two or more anions, including (C_8_H_18_N_2_)_7_(BiI_6_)_2_(Bi_2_I_9_)_2_·2I_3_ (Zhang *et al.*, 2018 ▸), and in inorganic compounds like Cs_3_Bi_2_I_9_ (Arakcheeva *et al.*, 2001 ▸).

## Optical properties and thermal stability   

**Figure 4:**
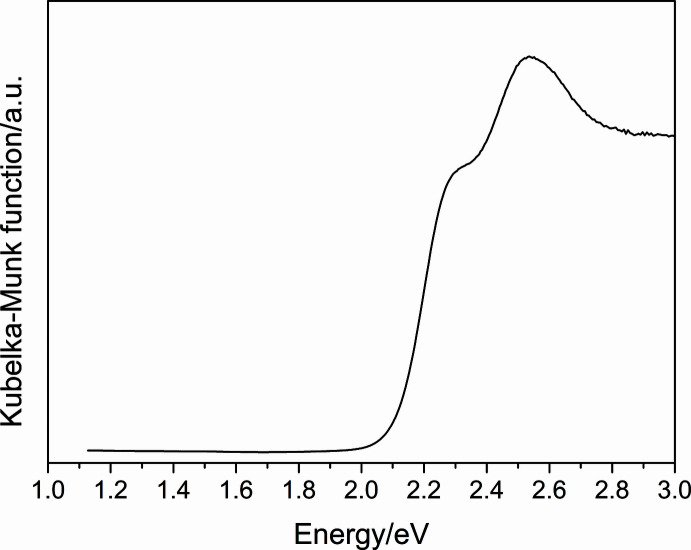
UV–vis diffuse reflectance for the title compound.

UV–vis diffuse reflectance data (Fig. 4 ▸) were collected on hand-picked single crystals, using a Perkin Elmer Lambda 35 UV–vis spectrometer. BaSO_4_ was used as a standard. The optical band gap, which was estimated from the absorption edge, is 2.1 eV. Thermogravimetric analysis (TGA) was carried out using a TA-TGA Q50 instrument. Measurements (see supporting information) carried out under a flowing nitro­gen atmosphere indicate that (C_9_H_7_N_2_)_3_Bi_2_I_9_ is stable up to 300°C.

## Synthesis and crystallization   

A mixture of BiI_3_ (1.1790 g, 2 mmol), KI (0.4490 g, 3 mmol), DBU (0.150 mL, 1 mmol) and ethanol (10 mL) was placed inside the Teflon liner of a 23 mL Parr autoclave. The autoclave was heated in an oven at 443 K for 6 days, using a heating and cooling rate of 0.1 K min^−1^. Following filtration, the collected solid product consisted of a mixture of red powder and crystals of the title compound. The powder X-ray diffraction pattern of the product (see supporting information), collected using a Bruker D8 Advance powder diffractometer (Cu *K*_α1_, λ = 1.5406 Å), was in excellent agreement with the simulated diffraction pattern, based on the single-crystal structure determination. Elemental analysis: Calculated values (%) for C_27_H_51_N_6_Bi_2_I_9_: C, 16.06; H, 2.55; N, 4.16. Found: C, 16.07; H, 2.42; N, 4.09. IR (ν_max_) cm^−1^: 2920, 2850 (C—H); 1640 (C=N).

## Refinement   

Crystal data, data collection and structure refinement details are summarized in Table 2 ▸. All hydrogen atoms were positioned geometrically, with C—H = 0.99 Å (for methyl­ene H atoms) and with N—H = 0.88 Å, and were refined with *U*_iso_(H) = 1.2*U*_eq_(C or N). The disordered DBUH^+^ cation has been refined using geometry (SAME) and *U*_ij_ restraints (SIMU and RIGU) implemented in *SHELXL*. The ratio between the site occupancies of the two positions was refined to 57.1 (13):42.9 (13)%. The maximum/minimum of the difference electron density is located 1.10 and 1.13 Å from Bi3 and Bi1, respectively. The electron density maxima and minima (2.22 and −2.57 e Å^−3^) close to the heavy bis­muth atoms can be ascribed to Fourier truncation ripples and/or non-ideal absorption correction.

## Supplementary Material

Crystal structure: contains datablock(s) I. DOI: 10.1107/S2056989021007799/jq2007sup1.cif

Structure factors: contains datablock(s) I. DOI: 10.1107/S2056989021007799/jq2007Isup3.hkl

Supporting information file. DOI: 10.1107/S2056989021007799/jq2007Isup4.cdx

Fingerprint plots, powder diffraction and TGA data. DOI: 10.1107/S2056989021007799/jq2007sup5.pdf

CCDC reference: 2100182

Additional supporting information:  crystallographic information; 3D view; checkCIF report

## Figures and Tables

**Table 1:** Hydrogen-bond geometry (Å, °)

*D*—H⋯*A*	*D*—H	H⋯*A*	*D*⋯*A*	*D*—H⋯*A*
N19—H19⋯I9^i^	0.88	2.81	3.632 (5)	156
N41—H41⋯I6^ii^	0.88	2.91	3.735 (13)	157
N41—H41⋯I8^ii^	0.88	3.21	3.663 (14)	115
N41*A*—H41*A*⋯I6^ii^	0.88	2.62	3.492 (15)	169

Symmetry codes: (i) x, -y+{\script{3\over 2}}, z+{\script{1\over 2}}; (ii) -x+1, y+{\script{1\over 2}}, -z+{\script{1\over 2}}.

**Table 2:** Experimental details

Crystal data	
Chemical formula	(C_9_H_17_N_2_)_3_[Bi_2_I_9_]
*M*_r_	2019.79
Crystal system, space group	Monoclinic, *P*2_1_/*c*
Temperature (K)	100
*a*, *b*, *c* (Å)	19.2590 (9), 12.5734 (3), 21.6767 (9)
β (°)	115.861 (5)
*V* (Å^3^)	4723.4 (4)
*Z*	4
Radiation type	Mo *K*α
μ (mm^−1^)	13.35
Crystal size (mm)	0.31 × 0.23 × 0.07

Data collection	
Diffractometer	XtaLAB Synergy, Dualflex, HyPix
Absorption correction	Multi-scan (*CrysAlis PRO*; Rigaku OD, 2019 ▸)
*T*_min_, *T*_max_	0.23, 1.00
No. of measured, independent and observed [*I* > 2σ(*I*)] reflections	38319, 13780, 11096
*R*_int_	0.034
(sin θ/λ)_max_ (Å^−1^)	0.714

Refinement	
*R*[*F*^2^ > 2σ(*F* ^2^)], *wR*(*F* ^2^), *S*	0.036, 0.086, 1.08
No. of reflections	13780
No. of parameters	497
No. of restraints	543
H-atom treatment	H-atom parameters constrained
Δρ_max_, Δρ_min_ (e Å^−3^)	2.22, −2.57

Computer programs: *CrysAlis PRO* (Rigaku OD, 2019 ▸), *SUPERFLIP* (Palatinus & Chapuis, 2007 ▸), *SHELXL2018/3* (Sheldrick, 2015 ▸), *ATOMS* (Dowty, 2020 ▸) and *ORIGIN* (Edwards, 2002 ▸).

## supplementary crystallographic information

### Crystal data

**Table:**

(C_9_H_17_N_2_)_3_[Bi_2_I_9_]	*F*(000) = 3592
*M**_r_* = 2019.79	*D*_x_ = 2.840 Mg m^−^^3^
Monoclinic, *P*2_1_/*c*	Mo *K*α radiation, λ = 0.71073 Å
*a* = 19.2590 (9) Å	Cell parameters from 25746 reflections
*b* = 12.5734 (3) Å	θ = 2–33°
*c* = 21.6767 (9) Å	µ = 13.35 mm^−^^1^
β = 115.861 (5)°	*T* = 100 K
*V* = 4723.4 (4) Å^3^	Plate, red
*Z* = 4	0.31 × 0.23 × 0.07 mm

### Data collection

**Table:**

XtaLAB Synergy, Dualflex, HyPix diffractometer	11096 reflections with *I* > 2σ(*I*)
ω scans	*R*_int_ = 0.034
Absorption correction: multi-scan (CrysAlisPro; Rigaku OD, 2019)	θ_max_ = 30.5°, θ_min_ = 2.4°
*T*_min_ = 0.23, *T*_max_ = 1.00	*h* = −27→27
38319 measured reflections	*k* = −13→17
13780 independent reflections	*l* = −30→29

### Refinement

**Table:**

Refinement on *F*^2^	543 restraints
Least-squares matrix: full	Hydrogen site location: inferred from neighbouring sites
*R*[*F*^2^ > 2σ(*F*^2^)] = 0.036	H-atom parameters constrained
*wR*(*F*^2^) = 0.086	*w* = 1/[σ^2^(*F*_o_^2^) + (0.0423*P*)^2^ + 1.9329*P*] where *P* = (*F*_o_^2^ + 2*F*_c_^2^)/3
*S* = 1.08	(Δ/σ)_max_ = 0.001
13780 reflections	Δρ_max_ = 2.22 e Å^−^^3^
497 parameters	Δρ_min_ = −2.57 e Å^−^^3^

### Special details

**Table:**

Geometry. All esds (except the esd in the dihedral angle between two l.s. planes) are estimated using the full covariance matrix. The cell esds are taken into account individually in the estimation of esds in distances, angles and torsion angles; correlations between esds in cell parameters are only used when they are defined by crystal symmetry. An approximate (isotropic) treatment of cell esds is used for estimating esds involving l.s. planes.

### Fractional atomic coordinates and isotropic or equivalent isotropic displacement parameters (Å^2^)

**Table:**

	*x*	*y*	*z*	*U*_iso_*/*U*_eq_	Occ. (<1)
Bi1	0.18727 (2)	0.75290 (2)	0.16730 (2)	0.01478 (5)	
Bi3	0.31503 (2)	0.48511 (2)	0.25029 (2)	0.01518 (5)	
I2	0.18622 (2)	0.60345 (3)	0.28735 (2)	0.01778 (8)	
I4	0.19072 (2)	0.52615 (3)	0.09942 (2)	0.01934 (8)	
I5	0.37010 (2)	0.72314 (3)	0.24092 (2)	0.02467 (9)	
I6	0.24567 (3)	0.27208 (3)	0.24502 (2)	0.02245 (9)	
I7	0.42044 (3)	0.48954 (3)	0.40035 (2)	0.02341 (9)	
I8	0.42451 (3)	0.39855 (3)	0.20120 (2)	0.02961 (10)	
I9	0.01394 (2)	0.74983 (3)	0.10767 (2)	0.01875 (8)	
I10	0.17891 (3)	0.84909 (3)	0.03995 (2)	0.02861 (10)	
I11	0.21740 (3)	0.95712 (3)	0.24184 (2)	0.02482 (9)	
N12	−0.0295 (3)	0.7605 (4)	0.3236 (2)	0.0195 (10)	
N19	−0.0307 (4)	0.7271 (4)	0.4272 (3)	0.0258 (12)	
H19	−0.027161	0.752892	0.466231	0.031*	
N23	0.4254 (4)	0.8196 (5)	0.5457 (3)	0.0360 (15)	
N30	0.3585 (4)	0.8010 (5)	0.4269 (3)	0.0396 (15)	
H30	0.359982	0.793472	0.387180	0.048*	
C13	−0.0306 (4)	0.8352 (5)	0.2708 (3)	0.0229 (13)	
H13A	−0.066990	0.893757	0.265999	0.028*	
H13B	−0.049722	0.797679	0.226308	0.028*	
C14	0.0479 (4)	0.8816 (5)	0.2876 (3)	0.0270 (15)	
H14A	0.048509	0.907961	0.244777	0.032*	
H14B	0.087103	0.824650	0.306148	0.032*	
C15	0.0701 (5)	0.9724 (5)	0.3392 (3)	0.0278 (15)	
H15A	0.033195	1.031594	0.319000	0.033*	
H15B	0.121944	0.998640	0.347472	0.033*	
C16	0.0714 (4)	0.9420 (5)	0.4083 (3)	0.0221 (13)	
H16A	0.108721	0.883316	0.428703	0.027*	
H16B	0.089943	1.003738	0.439604	0.027*	
C17	−0.0061 (4)	0.9077 (5)	0.4035 (3)	0.0238 (13)	
H17A	−0.006610	0.918916	0.448522	0.029*	
H17B	−0.046909	0.952911	0.369487	0.029*	
C18	−0.0238 (4)	0.7931 (5)	0.3832 (3)	0.0183 (11)	
C20	−0.0438 (5)	0.6139 (5)	0.4146 (3)	0.0389 (19)	
H20A	−0.099868	0.598977	0.392050	0.047*	
H20B	−0.020771	0.574505	0.458548	0.047*	
C21	−0.0072 (5)	0.5788 (5)	0.3692 (4)	0.0374 (18)	
H21A	0.049581	0.584182	0.394607	0.045*	
H21B	−0.020549	0.503466	0.355954	0.045*	
C22	−0.0347 (5)	0.6463 (5)	0.3062 (3)	0.0283 (15)	
H22A	−0.003083	0.631452	0.281397	0.034*	
H22B	−0.088950	0.627970	0.275421	0.034*	
C24	0.4962 (5)	0.8075 (6)	0.6123 (3)	0.0354 (18)	
H24A	0.518750	0.736198	0.613821	0.043*	
H24B	0.481188	0.811679	0.650508	0.043*	
C25	0.5580 (5)	0.8926 (6)	0.6230 (4)	0.0354 (17)	
H25A	0.532320	0.962602	0.608853	0.043*	
H25B	0.593503	0.896773	0.672436	0.043*	
C26	0.6046 (5)	0.8709 (6)	0.5832 (4)	0.0383 (18)	
H26A	0.633464	0.803665	0.600267	0.046*	
H26B	0.643041	0.928566	0.593145	0.046*	
C27	0.5574 (5)	0.8623 (5)	0.5048 (4)	0.0324 (16)	
H27A	0.529891	0.930270	0.487396	0.039*	
H27B	0.593515	0.852186	0.483876	0.039*	
C28	0.4989 (5)	0.7723 (5)	0.4815 (4)	0.0325 (17)	
H28A	0.486300	0.754439	0.433334	0.039*	
H28B	0.522400	0.708599	0.509803	0.039*	
C29	0.4259 (4)	0.7989 (5)	0.4868 (3)	0.0244 (14)	
C31	0.2843 (5)	0.8157 (7)	0.4294 (4)	0.0391 (18)	
H31A	0.265602	0.746710	0.438399	0.047*	
H31B	0.245533	0.843192	0.384877	0.047*	
C32	0.2947 (5)	0.8938 (6)	0.4860 (4)	0.0427 (19)	
H32A	0.306527	0.965247	0.473852	0.051*	
H32B	0.246359	0.898744	0.491447	0.051*	
C33	0.3583 (5)	0.8581 (6)	0.5509 (4)	0.0339 (16)	
H33A	0.373768	0.918258	0.583628	0.041*	
H33B	0.338628	0.800906	0.570454	0.041*	
N34	0.7076 (14)	0.569 (2)	0.4694 (6)	0.018 (2)	0.571 (13)
C35	0.7601 (11)	0.5636 (16)	0.5432 (7)	0.018 (3)	0.571 (13)
H35A	0.813909	0.558906	0.549092	0.022*	0.571 (13)
H35B	0.748948	0.498240	0.562769	0.022*	0.571 (13)
C36	0.7528 (11)	0.6591 (18)	0.5828 (10)	0.025 (3)	0.571 (13)
H36A	0.774972	0.639947	0.631953	0.030*	0.571 (13)
H36B	0.697209	0.673716	0.567634	0.030*	0.571 (13)
C37	0.7915 (15)	0.7619 (14)	0.5756 (10)	0.024 (3)	0.571 (13)
H37A	0.777857	0.819481	0.599494	0.029*	0.571 (13)
H37B	0.848090	0.751928	0.599731	0.029*	0.571 (13)
C38	0.7712 (11)	0.7984 (14)	0.5031 (10)	0.027 (3)	0.571 (13)
H38A	0.802108	0.862452	0.505442	0.032*	0.571 (13)
H38B	0.716141	0.819577	0.481252	0.032*	0.571 (13)
C39	0.7844 (9)	0.7154 (13)	0.4570 (8)	0.024 (3)	0.571 (13)
H39A	0.788929	0.752081	0.418453	0.028*	0.571 (13)
H39B	0.833494	0.677330	0.483789	0.028*	0.571 (13)
C40	0.7199 (12)	0.6369 (17)	0.4291 (7)	0.024 (4)	0.571 (13)
N41	0.6722 (8)	0.6439 (10)	0.3640 (6)	0.031 (3)	0.571 (13)
H41	0.682573	0.690516	0.338939	0.037*	0.571 (13)
C42	0.6022 (8)	0.5786 (10)	0.3302 (6)	0.031 (3)	0.571 (13)
H42A	0.590982	0.566712	0.281668	0.037*	0.571 (13)
H42B	0.557524	0.615825	0.331593	0.037*	0.571 (13)
C43	0.6149 (8)	0.4726 (8)	0.3674 (5)	0.027 (2)	0.571 (13)
H43A	0.565860	0.432290	0.349771	0.033*	0.571 (13)
H43B	0.653090	0.429953	0.358891	0.033*	0.571 (13)
C44	0.6436 (8)	0.4913 (12)	0.4427 (6)	0.020 (3)	0.571 (13)
H44A	0.600588	0.517838	0.451858	0.024*	0.571 (13)
H44B	0.661310	0.422934	0.467339	0.024*	0.571 (13)
N34A	0.7037 (19)	0.565 (3)	0.4637 (9)	0.020 (3)	0.429 (13)
C35A	0.7431 (15)	0.557 (2)	0.5392 (9)	0.018 (4)	0.429 (13)
H35C	0.798368	0.541270	0.553819	0.021*	0.429 (13)
H35D	0.720485	0.498255	0.554487	0.021*	0.429 (13)
C36A	0.7363 (15)	0.660 (2)	0.5737 (12)	0.020 (3)	0.429 (13)
H36C	0.741545	0.643507	0.620154	0.024*	0.429 (13)
H36D	0.684452	0.691150	0.546985	0.024*	0.429 (13)
C37A	0.7984 (19)	0.7428 (19)	0.5793 (12)	0.022 (4)	0.429 (13)
H37C	0.795358	0.804298	0.606510	0.026*	0.429 (13)
H37D	0.850144	0.710386	0.603988	0.026*	0.429 (13)
C38A	0.7887 (14)	0.7819 (16)	0.5091 (12)	0.022 (4)	0.429 (13)
H38C	0.826561	0.839271	0.515949	0.026*	0.429 (13)
H38D	0.736426	0.812666	0.484167	0.026*	0.429 (13)
C39A	0.7998 (11)	0.6933 (16)	0.4645 (11)	0.019 (3)	0.429 (13)
H39C	0.813384	0.726348	0.429756	0.022*	0.429 (13)
H39D	0.843318	0.647143	0.493943	0.022*	0.429 (13)
C40A	0.7293 (14)	0.626 (2)	0.4290 (8)	0.017 (3)	0.429 (13)
N41A	0.6957 (8)	0.6272 (12)	0.3614 (8)	0.018 (3)	0.429 (13)
H41A	0.717128	0.664894	0.340134	0.022*	0.429 (13)
C42A	0.6248 (11)	0.5690 (16)	0.3198 (8)	0.029 (3)	0.429 (13)
H42C	0.637673	0.498814	0.306758	0.035*	0.429 (13)
H42D	0.593866	0.609199	0.277302	0.035*	0.429 (13)
C43A	0.5791 (9)	0.5543 (12)	0.3608 (7)	0.027 (3)	0.429 (13)
H43C	0.558783	0.623766	0.366852	0.032*	0.429 (13)
H43D	0.534828	0.506463	0.335744	0.032*	0.429 (13)
C44A	0.6295 (13)	0.5075 (19)	0.4294 (8)	0.026 (4)	0.429 (13)
H44C	0.602112	0.510448	0.458819	0.031*	0.429 (13)
H44D	0.639682	0.431902	0.423618	0.031*	0.429 (13)

### Atomic displacement parameters (Å^2^)

**Table:**

	*U*^11^	*U*^22^	*U*^33^	*U*^12^	*U*^13^	*U*^23^
Bi1	0.01519 (11)	0.01399 (10)	0.01444 (10)	0.00064 (8)	0.00580 (9)	0.00318 (7)
Bi3	0.01585 (11)	0.01421 (10)	0.01379 (10)	0.00126 (8)	0.00490 (9)	−0.00033 (7)
I2	0.0225 (2)	0.01701 (17)	0.01585 (16)	0.00304 (14)	0.01026 (16)	0.00337 (12)
I4	0.0213 (2)	0.02260 (18)	0.01239 (16)	0.00069 (15)	0.00570 (16)	−0.00100 (13)
I5	0.0158 (2)	0.02024 (18)	0.0350 (2)	−0.00150 (15)	0.00837 (18)	0.00284 (15)
I6	0.0314 (2)	0.01434 (17)	0.01965 (18)	−0.00229 (15)	0.00928 (18)	−0.00232 (13)
I7	0.0242 (2)	0.02374 (19)	0.01498 (17)	0.00049 (16)	0.00181 (17)	0.00064 (14)
I8	0.0222 (2)	0.0374 (2)	0.0300 (2)	0.00495 (18)	0.01215 (19)	−0.00864 (17)
I9	0.01549 (19)	0.02433 (18)	0.01576 (17)	0.00086 (15)	0.00617 (16)	0.00014 (13)
I10	0.0275 (2)	0.0353 (2)	0.0234 (2)	−0.00061 (18)	0.01148 (19)	0.01407 (16)
I11	0.0246 (2)	0.01504 (17)	0.0310 (2)	0.00004 (16)	0.00855 (19)	−0.00205 (14)
N12	0.021 (3)	0.022 (2)	0.015 (2)	−0.003 (2)	0.008 (2)	−0.0051 (18)
N19	0.033 (3)	0.026 (3)	0.015 (2)	−0.010 (2)	0.007 (2)	−0.0008 (19)
N23	0.047 (4)	0.038 (3)	0.028 (3)	0.002 (3)	0.020 (3)	−0.001 (2)
N30	0.041 (4)	0.054 (4)	0.023 (3)	0.005 (3)	0.014 (3)	−0.008 (3)
C13	0.029 (4)	0.025 (3)	0.014 (3)	−0.004 (3)	0.010 (3)	−0.002 (2)
C14	0.037 (4)	0.027 (3)	0.021 (3)	−0.012 (3)	0.016 (3)	−0.008 (2)
C15	0.037 (4)	0.022 (3)	0.024 (3)	−0.006 (3)	0.013 (3)	−0.001 (2)
C16	0.029 (4)	0.019 (3)	0.010 (3)	−0.001 (3)	0.001 (3)	−0.003 (2)
C17	0.031 (4)	0.026 (3)	0.019 (3)	0.003 (3)	0.016 (3)	−0.004 (2)
C18	0.012 (3)	0.024 (3)	0.014 (3)	0.001 (2)	0.001 (2)	0.001 (2)
C20	0.055 (6)	0.032 (4)	0.019 (3)	−0.019 (4)	0.006 (4)	0.000 (3)
C21	0.049 (5)	0.016 (3)	0.034 (4)	−0.002 (3)	0.005 (4)	−0.003 (3)
C22	0.039 (4)	0.021 (3)	0.022 (3)	−0.004 (3)	0.011 (3)	−0.007 (2)
C24	0.047 (5)	0.038 (4)	0.022 (3)	0.017 (3)	0.016 (4)	0.011 (3)
C25	0.036 (5)	0.044 (4)	0.026 (3)	0.008 (3)	0.013 (3)	0.002 (3)
C26	0.039 (5)	0.041 (4)	0.038 (4)	0.017 (4)	0.019 (4)	0.006 (3)
C27	0.037 (5)	0.031 (3)	0.038 (4)	0.014 (3)	0.025 (4)	0.008 (3)
C28	0.048 (5)	0.028 (3)	0.026 (3)	0.017 (3)	0.019 (4)	0.007 (3)
C29	0.034 (4)	0.019 (3)	0.020 (3)	0.009 (3)	0.011 (3)	0.003 (2)
C31	0.031 (4)	0.059 (5)	0.027 (4)	−0.010 (4)	0.012 (3)	−0.010 (3)
C32	0.035 (5)	0.057 (5)	0.041 (4)	0.000 (4)	0.021 (4)	−0.013 (4)
C33	0.040 (5)	0.038 (4)	0.028 (4)	0.003 (3)	0.019 (4)	−0.003 (3)
N34	0.024 (5)	0.019 (4)	0.014 (4)	0.002 (4)	0.011 (4)	0.002 (3)
C35	0.021 (7)	0.017 (5)	0.018 (4)	0.001 (5)	0.010 (4)	0.005 (4)
C36	0.028 (7)	0.025 (5)	0.021 (5)	−0.001 (5)	0.010 (5)	−0.004 (4)
C37	0.024 (6)	0.022 (6)	0.027 (5)	0.002 (5)	0.011 (5)	−0.004 (5)
C38	0.025 (7)	0.022 (6)	0.030 (5)	−0.001 (5)	0.010 (5)	0.003 (4)
C39	0.026 (6)	0.028 (6)	0.016 (5)	−0.002 (5)	0.008 (5)	0.009 (4)
C40	0.026 (6)	0.027 (6)	0.017 (4)	0.001 (5)	0.009 (4)	0.007 (4)
N41	0.028 (6)	0.038 (6)	0.020 (4)	−0.010 (5)	0.004 (4)	0.010 (4)
C42	0.028 (6)	0.034 (5)	0.015 (5)	−0.002 (5)	−0.004 (4)	0.007 (4)
C43	0.028 (5)	0.029 (5)	0.019 (4)	0.002 (4)	0.004 (4)	0.004 (3)
C44	0.018 (5)	0.022 (5)	0.017 (4)	0.004 (4)	0.005 (4)	0.003 (4)
N34A	0.025 (5)	0.018 (6)	0.014 (4)	−0.001 (5)	0.007 (4)	0.003 (4)
C35A	0.020 (8)	0.018 (6)	0.015 (4)	−0.004 (6)	0.007 (5)	−0.003 (4)
C36A	0.023 (7)	0.024 (5)	0.016 (6)	−0.005 (6)	0.012 (6)	−0.003 (5)
C37A	0.027 (7)	0.017 (7)	0.024 (5)	−0.005 (6)	0.013 (5)	−0.007 (5)
C38A	0.022 (8)	0.013 (6)	0.028 (6)	0.004 (6)	0.009 (6)	0.005 (5)
C39A	0.020 (7)	0.017 (7)	0.020 (6)	0.001 (5)	0.009 (5)	0.005 (5)
C40A	0.018 (6)	0.018 (6)	0.017 (4)	0.006 (5)	0.009 (4)	0.002 (4)
N41A	0.018 (6)	0.022 (5)	0.015 (4)	0.003 (5)	0.007 (4)	0.000 (4)
C42A	0.028 (7)	0.043 (7)	0.014 (5)	−0.006 (5)	0.007 (4)	−0.004 (5)
C43A	0.023 (6)	0.036 (6)	0.019 (5)	−0.005 (5)	0.007 (4)	0.003 (5)
C44A	0.030 (7)	0.022 (7)	0.020 (5)	−0.007 (6)	0.005 (5)	0.002 (5)

### Geometric parameters (Å, º)

**Table:**

Bi1—I10	2.9532 (4)	C31—H31B	0.9900
Bi1—I11	2.9534 (4)	C32—C33	1.476 (11)
Bi1—I9	3.0085 (5)	C32—H32A	0.9900
Bi1—I5	3.1911 (5)	C32—H32B	0.9900
Bi1—I2	3.2169 (4)	C33—H33A	0.9900
Bi1—I4	3.2224 (4)	C33—H33B	0.9900
Bi3—I8	2.9530 (5)	N34—C40	1.318 (11)
Bi3—I6	2.9730 (4)	N34—C35	1.475 (12)
Bi3—I7	2.9908 (5)	N34—C44	1.475 (13)
Bi3—I4	3.1405 (5)	C35—C36	1.516 (12)
Bi3—I5	3.2111 (4)	C35—H35A	0.9900
Bi3—I2	3.2788 (4)	C35—H35B	0.9900
N12—C18	1.313 (7)	C36—C37	1.533 (13)
N12—C13	1.473 (7)	C36—H36A	0.9900
N12—C22	1.477 (7)	C36—H36B	0.9900
N19—C18	1.314 (7)	C37—C38	1.516 (13)
N19—C20	1.451 (8)	C37—H37A	0.9900
N19—H19	0.8800	C37—H37B	0.9900
N23—C29	1.308 (7)	C38—C39	1.543 (13)
N23—C33	1.430 (10)	C38—H38A	0.9900
N23—C24	1.500 (10)	C38—H38B	0.9900
N30—C29	1.379 (9)	C39—C40	1.493 (13)
N30—C31	1.465 (10)	C39—H39A	0.9900
N30—H30	0.8800	C39—H39B	0.9900
C13—C14	1.510 (9)	C40—N41	1.308 (12)
C13—H13A	0.9900	N41—C42	1.472 (14)
C13—H13B	0.9900	N41—H41	0.8800
C14—C15	1.523 (8)	C42—C43	1.521 (14)
C14—H14A	0.9900	C42—H42A	0.9900
C14—H14B	0.9900	C42—H42B	0.9900
C15—C16	1.536 (8)	C43—C44	1.497 (16)
C15—H15A	0.9900	C43—H43A	0.9900
C15—H15B	0.9900	C43—H43B	0.9900
C16—C17	1.512 (9)	C44—H44A	0.9900
C16—H16A	0.9900	C44—H44B	0.9900
C16—H16B	0.9900	N34A—C40A	1.319 (13)
C17—C18	1.502 (8)	N34A—C35A	1.476 (13)
C17—H17A	0.9900	N34A—C44A	1.477 (15)
C17—H17B	0.9900	C35A—C36A	1.525 (14)
C20—C21	1.505 (11)	C35A—H35C	0.9900
C20—H20A	0.9900	C35A—H35D	0.9900
C20—H20B	0.9900	C36A—C37A	1.549 (16)
C21—C22	1.495 (9)	C36A—H36C	0.9900
C21—H21A	0.9900	C36A—H36D	0.9900
C21—H21B	0.9900	C37A—C38A	1.530 (14)
C22—H22A	0.9900	C37A—H37C	0.9900
C22—H22B	0.9900	C37A—H37D	0.9900
C24—C25	1.541 (11)	C38A—C39A	1.551 (15)
C24—H24A	0.9900	C38A—H38C	0.9900
C24—H24B	0.9900	C38A—H38D	0.9900
C25—C26	1.517 (10)	C39A—C40A	1.492 (14)
C25—H25A	0.9900	C39A—H39C	0.9900
C25—H25B	0.9900	C39A—H39D	0.9900
C26—C27	1.542 (11)	C40A—N41A	1.317 (13)
C26—H26A	0.9900	N41A—C42A	1.462 (15)
C26—H26B	0.9900	N41A—H41A	0.8800
C27—C28	1.519 (11)	C42A—C43A	1.510 (16)
C27—H27A	0.9900	C42A—H42C	0.9900
C27—H27B	0.9900	C42A—H42D	0.9900
C28—C29	1.497 (10)	C43A—C44A	1.497 (18)
C28—H28A	0.9900	C43A—H43C	0.9900
C28—H28B	0.9900	C43A—H43D	0.9900
C31—C32	1.515 (10)	C44A—H44C	0.9900
C31—H31A	0.9900	C44A—H44D	0.9900
			
I10—Bi1—I11	94.180 (13)	C32—C31—H31A	109.9
I10—Bi1—I9	90.328 (14)	N30—C31—H31B	109.9
I11—Bi1—I9	99.358 (13)	C32—C31—H31B	109.9
I10—Bi1—I5	96.536 (14)	H31A—C31—H31B	108.3
I11—Bi1—I5	85.283 (13)	C33—C32—C31	109.6 (7)
I9—Bi1—I5	171.439 (12)	C33—C32—H32A	109.7
I10—Bi1—I2	168.049 (13)	C31—C32—H32A	109.7
I11—Bi1—I2	97.725 (12)	C33—C32—H32B	109.7
I9—Bi1—I2	86.722 (12)	C31—C32—H32B	109.7
I5—Bi1—I2	85.519 (12)	H32A—C32—H32B	108.2
I10—Bi1—I4	86.507 (12)	N23—C33—C32	115.5 (6)
I11—Bi1—I4	168.754 (14)	N23—C33—H33A	108.4
I9—Bi1—I4	91.859 (12)	C32—C33—H33A	108.4
I5—Bi1—I4	83.488 (12)	N23—C33—H33B	108.4
I2—Bi1—I4	82.022 (10)	C32—C33—H33B	108.4
I8—Bi3—I6	91.922 (13)	H33A—C33—H33B	107.5
I8—Bi3—I7	98.404 (15)	C40—N34—C35	120.7 (11)
I6—Bi3—I7	97.988 (12)	C40—N34—C44	122.0 (10)
I8—Bi3—I4	91.439 (13)	C35—N34—C44	117.2 (10)
I6—Bi3—I4	88.991 (12)	N34—C35—C36	113.0 (13)
I7—Bi3—I4	167.695 (12)	N34—C35—H35A	109.0
I8—Bi3—I5	90.760 (13)	C36—C35—H35A	109.0
I6—Bi3—I5	173.004 (14)	N34—C35—H35B	109.0
I7—Bi3—I5	88.005 (12)	C36—C35—H35B	109.0
I4—Bi3—I5	84.482 (12)	H35A—C35—H35B	107.8
I8—Bi3—I2	172.288 (13)	C35—C36—C37	116.2 (12)
I6—Bi3—I2	92.465 (12)	C35—C36—H36A	108.2
I7—Bi3—I2	87.263 (12)	C37—C36—H36A	108.2
I4—Bi3—I2	82.301 (11)	C35—C36—H36B	108.2
I5—Bi3—I2	84.183 (11)	C37—C36—H36B	108.2
Bi1—I2—Bi3	78.144 (9)	H36A—C36—H36B	107.4
Bi3—I4—Bi1	80.095 (10)	C38—C37—C36	116.3 (13)
Bi1—I5—Bi3	79.516 (11)	C38—C37—H37A	108.2
C18—N12—C13	122.1 (5)	C36—C37—H37A	108.2
C18—N12—C22	121.5 (5)	C38—C37—H37B	108.2
C13—N12—C22	116.4 (4)	C36—C37—H37B	108.2
C18—N19—C20	123.2 (5)	H37A—C37—H37B	107.4
C18—N19—H19	118.4	C37—C38—C39	115.2 (12)
C20—N19—H19	118.4	C37—C38—H38A	108.5
C29—N23—C33	122.4 (7)	C39—C38—H38A	108.5
C29—N23—C24	121.9 (6)	C37—C38—H38B	108.5
C33—N23—C24	115.6 (5)	C39—C38—H38B	108.5
C29—N30—C31	120.1 (6)	H38A—C38—H38B	107.5
C29—N30—H30	119.9	C40—C39—C38	111.4 (12)
C31—N30—H30	119.9	C40—C39—H39A	109.3
N12—C13—C14	112.7 (5)	C38—C39—H39A	109.3
N12—C13—H13A	109.0	C40—C39—H39B	109.3
C14—C13—H13A	109.0	C38—C39—H39B	109.3
N12—C13—H13B	109.0	H39A—C39—H39B	108.0
C14—C13—H13B	109.0	N41—C40—N34	120.9 (11)
H13A—C13—H13B	107.8	N41—C40—C39	117.4 (11)
C13—C14—C15	113.4 (5)	N34—C40—C39	121.4 (11)
C13—C14—H14A	108.9	C40—N41—C42	124.0 (11)
C15—C14—H14A	108.9	C40—N41—H41	118.0
C13—C14—H14B	108.9	C42—N41—H41	118.0
C15—C14—H14B	108.9	N41—C42—C43	108.9 (10)
H14A—C14—H14B	107.7	N41—C42—H42A	109.9
C14—C15—C16	114.2 (5)	C43—C42—H42A	109.9
C14—C15—H15A	108.7	N41—C42—H42B	109.9
C16—C15—H15A	108.7	C43—C42—H42B	109.9
C14—C15—H15B	108.7	H42A—C42—H42B	108.3
C16—C15—H15B	108.7	C44—C43—C42	109.7 (10)
H15A—C15—H15B	107.6	C44—C43—H43A	109.7
C17—C16—C15	114.2 (5)	C42—C43—H43A	109.7
C17—C16—H16A	108.7	C44—C43—H43B	109.7
C15—C16—H16A	108.7	C42—C43—H43B	109.7
C17—C16—H16B	108.7	H43A—C43—H43B	108.2
C15—C16—H16B	108.7	N34—C44—C43	112.3 (10)
H16A—C16—H16B	107.6	N34—C44—H44A	109.2
C18—C17—C16	112.6 (5)	C43—C44—H44A	109.2
C18—C17—H17A	109.1	N34—C44—H44B	109.2
C16—C17—H17A	109.1	C43—C44—H44B	109.2
C18—C17—H17B	109.1	H44A—C44—H44B	107.9
C16—C17—H17B	109.1	C40A—N34A—C35A	121.9 (14)
H17A—C17—H17B	107.8	C40A—N34A—C44A	121.3 (13)
N12—C18—N19	121.7 (5)	C35A—N34A—C44A	116.4 (14)
N12—C18—C17	120.0 (5)	N34A—C35A—C36A	112.3 (17)
N19—C18—C17	118.3 (5)	N34A—C35A—H35C	109.1
N19—C20—C21	108.6 (6)	C36A—C35A—H35C	109.1
N19—C20—H20A	110.0	N34A—C35A—H35D	109.1
C21—C20—H20A	110.0	C36A—C35A—H35D	109.1
N19—C20—H20B	110.0	H35C—C35A—H35D	107.9
C21—C20—H20B	110.0	C35A—C36A—C37A	112.2 (16)
H20A—C20—H20B	108.4	C35A—C36A—H36C	109.2
C22—C21—C20	110.6 (6)	C37A—C36A—H36C	109.2
C22—C21—H21A	109.5	C35A—C36A—H36D	109.2
C20—C21—H21A	109.5	C37A—C36A—H36D	109.2
C22—C21—H21B	109.5	H36C—C36A—H36D	107.9
C20—C21—H21B	109.5	C38A—C37A—C36A	112.5 (17)
H21A—C21—H21B	108.1	C38A—C37A—H37C	109.1
N12—C22—C21	111.1 (5)	C36A—C37A—H37C	109.1
N12—C22—H22A	109.4	C38A—C37A—H37D	109.1
C21—C22—H22A	109.4	C36A—C37A—H37D	109.1
N12—C22—H22B	109.4	H37C—C37A—H37D	107.8
C21—C22—H22B	109.4	C37A—C38A—C39A	113.5 (14)
H22A—C22—H22B	108.0	C37A—C38A—H38C	108.9
N23—C24—C25	113.1 (5)	C39A—C38A—H38C	108.9
N23—C24—H24A	109.0	C37A—C38A—H38D	108.9
C25—C24—H24A	109.0	C39A—C38A—H38D	108.9
N23—C24—H24B	109.0	H38C—C38A—H38D	107.7
C25—C24—H24B	109.0	C40A—C39A—C38A	112.6 (15)
H24A—C24—H24B	107.8	C40A—C39A—H39C	109.1
C26—C25—C24	113.6 (6)	C38A—C39A—H39C	109.1
C26—C25—H25A	108.9	C40A—C39A—H39D	109.1
C24—C25—H25A	108.9	C38A—C39A—H39D	109.1
C26—C25—H25B	108.9	H39C—C39A—H39D	107.8
C24—C25—H25B	108.9	N41A—C40A—N34A	120.9 (13)
H25A—C25—H25B	107.7	N41A—C40A—C39A	117.7 (14)
C25—C26—C27	115.3 (7)	N34A—C40A—C39A	121.3 (13)
C25—C26—H26A	108.4	C40A—N41A—C42A	123.8 (13)
C27—C26—H26A	108.4	C40A—N41A—H41A	118.1
C25—C26—H26B	108.4	C42A—N41A—H41A	118.1
C27—C26—H26B	108.4	N41A—C42A—C43A	108.9 (12)
H26A—C26—H26B	107.5	N41A—C42A—H42C	109.9
C28—C27—C26	114.4 (6)	C43A—C42A—H42C	109.9
C28—C27—H27A	108.7	N41A—C42A—H42D	109.9
C26—C27—H27A	108.7	C43A—C42A—H42D	109.9
C28—C27—H27B	108.7	H42C—C42A—H42D	108.3
C26—C27—H27B	108.7	C44A—C43A—C42A	110.0 (15)
H27A—C27—H27B	107.6	C44A—C43A—H43C	109.7
C29—C28—C27	113.2 (5)	C42A—C43A—H43C	109.7
C29—C28—H28A	108.9	C44A—C43A—H43D	109.7
C27—C28—H28A	108.9	C42A—C43A—H43D	109.7
C29—C28—H28B	108.9	H43C—C43A—H43D	108.2
C27—C28—H28B	108.9	N34A—C44A—C43A	112.0 (14)
H28A—C28—H28B	107.7	N34A—C44A—H44C	109.2
N23—C29—N30	120.6 (6)	C43A—C44A—H44C	109.2
N23—C29—C28	121.7 (6)	N34A—C44A—H44D	109.2
N30—C29—C28	117.6 (5)	C43A—C44A—H44D	109.2
N30—C31—C32	108.9 (6)	H44C—C44A—H44D	107.9
N30—C31—H31A	109.9		
			
C18—N12—C13—C14	74.6 (7)	C40—N34—C35—C36	71 (3)
C22—N12—C13—C14	−105.1 (6)	C44—N34—C35—C36	−111 (2)
N12—C13—C14—C15	−79.8 (7)	N34—C35—C36—C37	−77.1 (19)
C13—C14—C15—C16	58.5 (8)	C35—C36—C37—C38	52 (2)
C14—C15—C16—C17	−62.5 (8)	C36—C37—C38—C39	−55 (2)
C15—C16—C17—C18	82.5 (6)	C37—C38—C39—C40	80.1 (16)
C13—N12—C18—N19	173.0 (6)	C35—N34—C40—N41	−178 (2)
C22—N12—C18—N19	−7.4 (10)	C44—N34—C40—N41	5 (5)
C13—N12—C18—C17	−9.3 (9)	C35—N34—C40—C39	−3 (4)
C22—N12—C18—C17	170.3 (6)	C44—N34—C40—C39	179 (2)
C20—N19—C18—N12	1.0 (11)	C38—C39—C40—N41	108 (2)
C20—N19—C18—C17	−176.7 (7)	C38—C39—C40—N34	−67 (3)
C16—C17—C18—N12	−61.7 (8)	N34—C40—N41—C42	0 (4)
C16—C17—C18—N19	116.1 (6)	C39—C40—N41—C42	−174.6 (15)
C18—N19—C20—C21	29.8 (10)	C40—N41—C42—C43	−29 (2)
N19—C20—C21—C22	−53.0 (8)	N41—C42—C43—C44	50.9 (15)
C18—N12—C22—C21	−18.5 (9)	C40—N34—C44—C43	21 (4)
C13—N12—C22—C21	161.1 (6)	C35—N34—C44—C43	−156.7 (18)
C20—C21—C22—N12	48.4 (9)	C42—C43—C44—N34	−48 (2)
C29—N23—C24—C25	70.8 (8)	C40A—N34A—C35A—C36A	69 (4)
C33—N23—C24—C25	−107.4 (7)	C44A—N34A—C35A—C36A	−105 (3)
N23—C24—C25—C26	−78.4 (8)	N34A—C35A—C36A—C37A	−84 (2)
C24—C25—C26—C27	58.7 (8)	C35A—C36A—C37A—C38A	65 (3)
C25—C26—C27—C28	−61.5 (8)	C36A—C37A—C38A—C39A	−64 (3)
C26—C27—C28—C29	80.3 (7)	C37A—C38A—C39A—C40A	81 (2)
C33—N23—C29—N30	−8.1 (10)	C35A—N34A—C40A—N41A	176 (3)
C24—N23—C29—N30	173.8 (6)	C44A—N34A—C40A—N41A	−10 (6)
C33—N23—C29—C28	171.9 (6)	C35A—N34A—C40A—C39A	0 (6)
C24—N23—C29—C28	−6.2 (10)	C44A—N34A—C40A—C39A	173 (3)
C31—N30—C29—N23	−6.3 (10)	C38A—C39A—C40A—N41A	117 (2)
C31—N30—C29—C28	173.7 (6)	C38A—C39A—C40A—N34A	−66 (4)
C27—C28—C29—N23	−62.7 (8)	N34A—C40A—N41A—C42A	6 (5)
C27—C28—C29—N30	117.3 (7)	C39A—C40A—N41A—C42A	−177.6 (18)
C29—N30—C31—C32	37.4 (9)	C40A—N41A—C42A—C43A	26 (3)
N30—C31—C32—C33	−53.5 (9)	N41A—C42A—C43A—C44A	−51 (2)
C29—N23—C33—C32	−12.0 (10)	C40A—N34A—C44A—C43A	−18 (5)
C24—N23—C33—C32	166.2 (6)	C35A—N34A—C44A—C43A	156 (3)
C31—C32—C33—N23	42.8 (9)	C42A—C43A—C44A—N34A	49 (3)

### Hydrogen-bond geometry (Å, º)

**Table:**

*D*—H···*A*	*D*—H	H···*A*	*D*···*A*	*D*—H···*A*
N19—H19···I9^i^	0.88	2.81	3.632 (5)	156
N41—H41···I6^ii^	0.88	2.91	3.735 (13)	157
N41—H41···I8^ii^	0.88	3.21	3.663 (14)	115
N41*A*—H41*A*···I6^ii^	0.88	2.62	3.492 (15)	169

Symmetry codes: (i) *x*, −*y*+3/2, *z*+1/2; (ii) −*x*+1, *y*+1/2, −*z*+1/2.
