# Supplementary material for: Crystal structure of (C9H17N2)3[Bi2I9]
Source: Acta Crystallogr E Crystallogr Commun. 2021 Aug 6;77(Pt 9):899–902. doi: 10.1107/S2056989021007799 (PMC8423012; doi:10.1107/S2056989021007799)
Supplement: Supplementary file 4 [file e-77-00899-sup5.pdf]

Supporting Information

Crystal structure of $(C_9H_{17}N_2)_3Bi_2I_9$

Zoe James, Yunhe Cai and Paz Vaqueiro

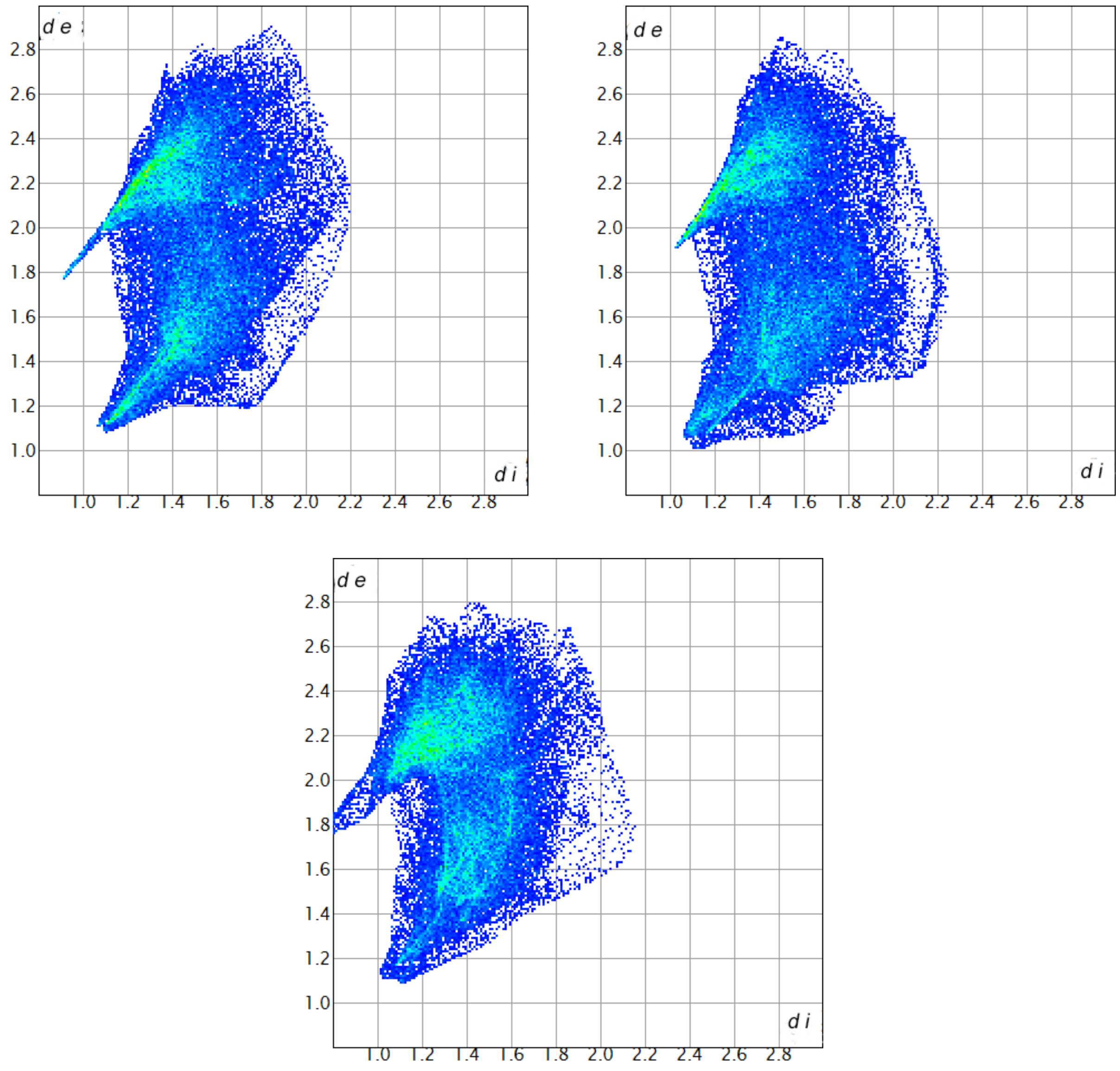

Figure S1. The overall fingerprint plots for the three crystallographically-independent $DBUH^+$ cations.

(a)

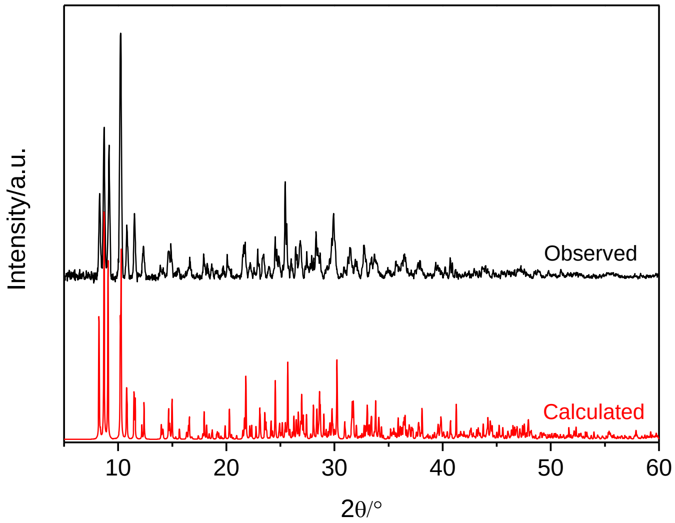

(b)

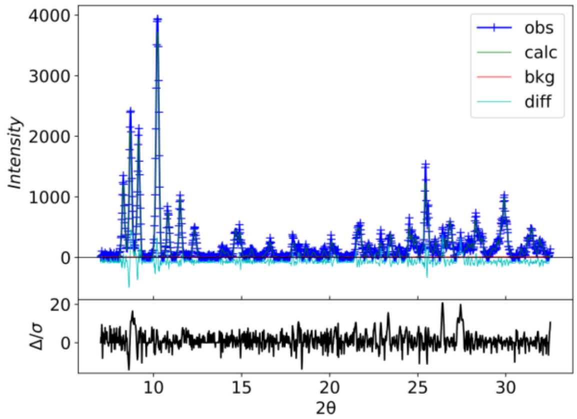

Figure S2. (a) Experimental (black line) and calculated (red line) powder X-ray diffraction data for $(C_9H_{17}N_2)_3Bi_2I_9$ (b) Pawley refinement using powder X-ray diffraction data for $(C_9H_{17}N_2)_3Bi_2I_9$, which resulted in the following lattice parameters: *a* = 19.374(7), *b* = 12.629(1), *c* = 21.510(8) Å, *β* = 116.08(1)°.

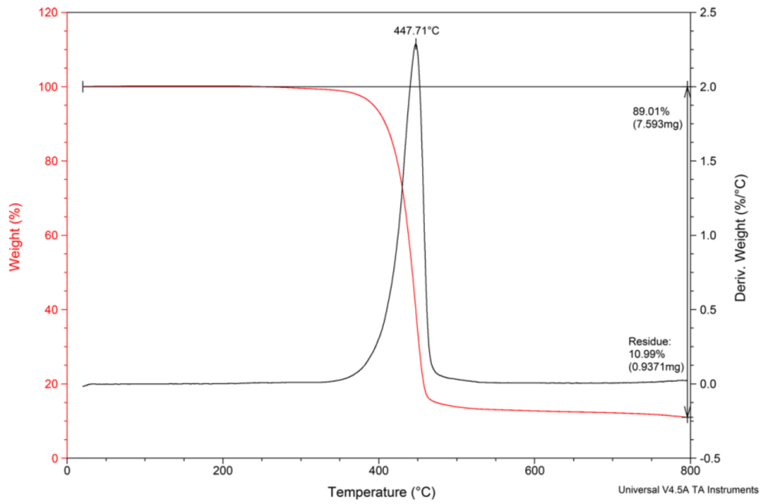

Figure S3. Thermogravimetric data for $(C_9H_{17}N_2)_3Bi_2I_9$, collected under a N_2 atmosphere.
